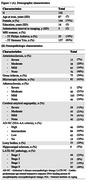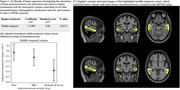# Brain arteriolosclerosis severity is associated with lower cortical volume in a community cohort of older adults

**DOI:** 10.1002/alz70855_100588

**Published:** 2025-12-23

**Authors:** Ana Tomash, Md Tahmid Yasar, David A. A. Bennett, Julie A Schneider, Konstantinos Arfanakis

**Affiliations:** ^1^ Illinois Institute of Technology, Chicago, IL, USA; ^2^ Rush Alzheimer's Disease Center, Chicago, IL, USA; ^3^ Rush Alzheimer's Disease Center, Rush University Medical Center, Chicago, IL, USA

## Abstract

**Background:**

Brain arteriolosclerosis, characterized by vessel wall thickening and arteriolar narrowing, is a primary pathology of cerebral small vessel disease and is prevalent in older adults. Greater severity is observed in women and black individuals, and the condition is associated with reduced cognitive and motor abilities and increased dementia risk. Despite its widespread occurrence, the impact of arteriolosclerosis on brain macrostructure remains unexplored. This study examined the relationship between brain arteriolosclerosis and in‐vivo subcortical and cortical volumes in a large cohort of community‐based older adults.

**Methods:**

This study included 192 older adults from four longitudinal aging cohort studies (Rush Memory and Aging Project, Religious Orders Study, Minority Aging Research Study, and the Clinical Core of the Rush Alzheimer's Disease Research Center). In‐vivo 3D T1‐weighted MPRAGE imaging was performed using 3T MRI scanners (Figure 1A). Subcortical and cortical brain volumes were segmented using multi‐atlas segmentation and then normalized by the intracranial volume. A board‐certified neuropathologist examined postmortem brains. The severity of arteriolosclerosis was categorized as none, mild, moderate, or severe. Other neuropathologies assessed included atherosclerosis, cerebral amyloid angiopathy, gross and microscopic infarcts, Alzheimer's pathology, Lewy bodies, limbic‐predominant age‐related TDP‐43 encephalopathy neuropathological change (LATE‐NC), and hippocampal sclerosis (Figure 1B). Linear regression assessed associations between arteriolosclerosis severity and normalized brain volumes, controlling for demographics, antemortem interval, scanner, and other neuropathologies (Figure 1). Statistical analysis was conducted using FSL's PALM tool, with 10,000 permutations. After correcting for multiple testing using the family‐wise error rate (FWER) significance was set at *p* <0.05. Regions exhibiting significant associations were overlaid on the MIITRA atlas.

**Results:**

Higher severity of arteriolosclerosis was linked with lower volume in the middle temporal gyrus, independent of demographics and other neuropathologies (Figure 2). The abnormality in the volume started with mild arteriolosclerosis and became more significant for moderate and severe arteriolosclerosis (Figure 2B). This study, conducted in a large cohort of community‐based older adults, provides robust evidence that arteriolosclerosis is also associated with neurodegenerative changes in the temporal lobe.

**Conclusion:**

This study integrates in‐vivo MRI with detailed neuropathological assessment and demonstrates that brain arteriolosclerosis is associated with middle temporal gyrus volume loss, independent of other pathologies.